# The effect of population inversion saturation on the transverse mode instability threshold in high power fiber laser oscillators

**DOI:** 10.1038/s41598-021-00400-9

**Published:** 2021-10-26

**Authors:** Ali Roohforouz, Reza Eyni Chenar, Reza Rezaei-Nasirabad, Saeed Azizi, Kamran Hejaz, Ali Hamedani Golshan, Ali Abedinajafi, Vahid Vatani, Seyed Hassan Nabavi

**Affiliations:** Iranian National Center for Laser Science and Technology, Tehran, Iran

**Keywords:** Fibre lasers, Nonlinear optics

## Abstract

To achieve a 3.02 kW Yb-doped fiber laser oscillator, the behavior of transverse mode instability (TMI) is experimentally studied in different pumping configurations; co, hybrid, counter, and bidirectional. A comparative analysis showed that population inversion saturation has a substantial impact on TMI threshold enhancement in high power fiber oscillators. Monitoring the dynamic power exchange of fundamental mode and higher-order mode of laser output beam indicates that in a hybrid pumping scheme, simultaneous pumping with two different wavelengths enhances the TMI threshold to a great stand. Moreover, injecting a few watts of pumping light in the counter direction mitigates the TMI caused by pumping in the co-direction. Calculation of population inversion in different pumping configurations using simulation shows that higher population inversion saturation leads to increasing the TMI threshold.

## Introduction

High power fiber lasers are attracting a great deal of attention thanks to the groundbreaking strategies in controlling destructive nonlinearities and progress in high power fiber element technologies. High power fiber lasers are usually manufactured in two configurations, master oscillator power amplifier(MOPA) and fiber laser oscillator. Although the research on TMI and SRS are mainly focused on MOPA. In comparison to amplifiers, high-power monolithic fiber laser oscillators enjoy the advantages of simpler structure, easier manipulation, and less sensitivity against backward coupled light in industrial applications. To keep the increasing pace of the demand for their applications, people are removing the barriers to the power scaling of fiber laser systems. The power scaling of fiber amplifiers and oscillators has been faced with two major problems; transverse mode instability (TMI)^1^ and stimulated Raman scattering (SRS)^2,3^. These destructive effects create serious constraints on the efficiency and beam quality of high-power fiber lasers in the last decade.

Since the first publication of TMI in fiber laser systems^4^, lots of theoretical and experimental studies have been reported on TMI mitigation strategies^5–10^. Some conventional ways to avoid the TMI in high-power laser systems are to use extremely low NA fibers^11^, tight coiling^12,13^ and controlling the heat load of the gain fiber by making a shift between the pumping wavelength and the gain fiber’s peak absorption wavelength^14^. Concerning the SRS, enlargement of the fiber mode area^15^, decreasing the effective fiber length, and broadening the bandwidth of fiber Bragg gratings (FBGs)^16^ are the most common suppressing methods. Moreover, there is a kind of TMI induced by the SRS effect in high power fiber amplifiers^17^ which is mitigated by SRS suppression^18^.

To enhance the TMI threshold of oscillators the effect of pumping direction and wavelength have been noticed^12,14,19,20^. The advantage of counter-pumping to co-pumping in TMI threshold enhancement has been shown theoretically and experimentally^21,22^. Regarding the bi-directional pumping scheme, the TMI threshold is higher than co and counter pumping^12^.

In this paper, the effect of population inversion saturation on the TMI threshold is investigated in different pumping schemes. The TMI threshold in co, hybrid, counter and bidirectional pumping methods have been investigated quantitatively, and the results are discussed analytically. The inversion level of each configuration is calculated by Liekki application designer V3.3 software to compare these different pumping methods from the viewpoint of gain saturation. Moreover, The beneficial effect of hybrid pumping by 971 and 976 nm wavelengths on the TMI threshold is advised. Here reported achievements to pave the way for a better understanding of the TMI phenomenon and can lead to an increase of TMI threshold and substantial power scaling of fiber oscillators. Finally, the authors present a 3.02 kW single-mode output power with no evidence of TMI and SRS using a bi-directional pumping scheme.

**Figure 1:**
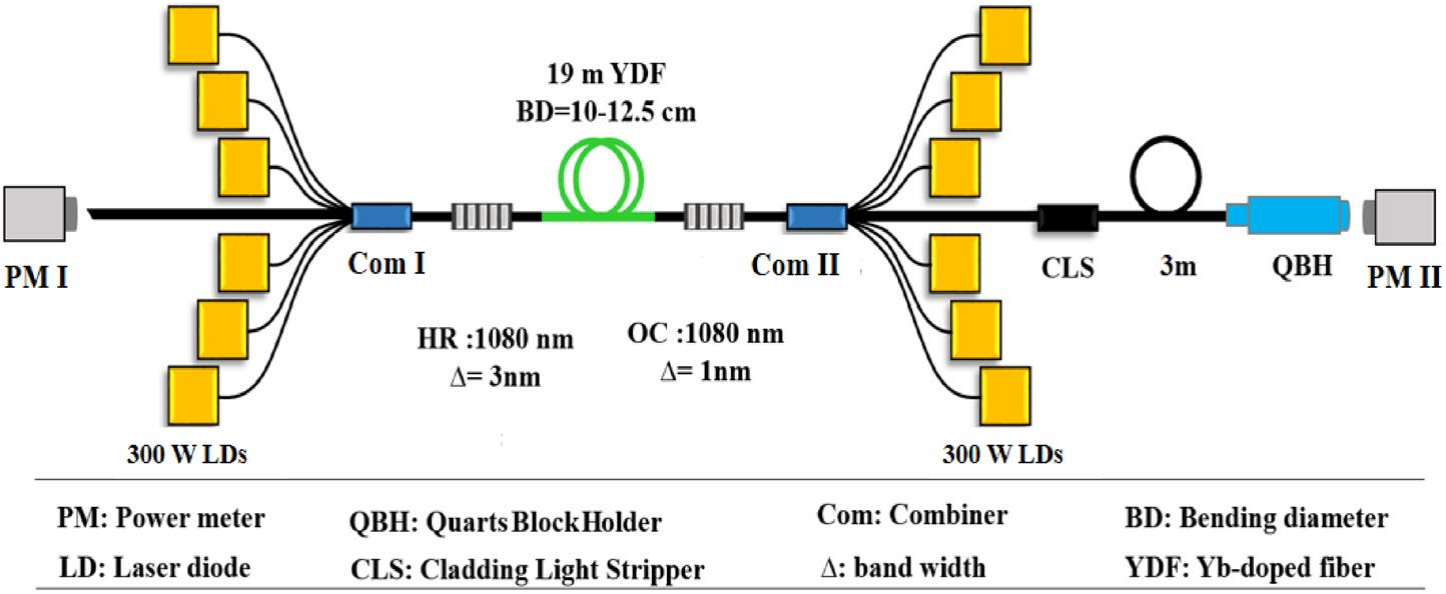
Schematic diagram of the bidirectional pumped configuration

## Experimental setup

Schematic setup of bidirectional pumped fiber laser oscillator is shown in Fig. 1. Twelve 300 W laser diodes (LDs) with 976 nm central wavelength, 1 nm tolerance of central wavelength and 3 nm spectral bandwidth are used to provide 3.6 kW pumping power. Half of the diodes launch pump light in the co-direction and the remained in the counter-direction.

Based on simulation results, 19 m 20/400 μm double cladding Yb-doped fiber (YDF) with a numerical aperture of $NA=0.065/0.46$ (core/clad) and an absorption coefficient of 1.26 dB/m at 976 nm is employed as the gain medium. The gain fiber is coiled in a symmetrical racetrack scheme with a minimum bending diameter of 10 cm at the beginning of the gain medium and a maximum bending diameter of 12.5 cm. The high reflection fiber Bragg grating (HR FBG) provides a reflectivity of 99% at the center wavelength of 1080 nm with a 3 dB bandwidth of 3 nm, while the output coupler (OC) FBG has a reflectivity of  10% with 3 dB bandwidth of 1 nm.

The undesirable cladding light which degrades the laser beam quality and may damage the output fiber connector is removed by a cladding light stripper (CLS) that is spliced to the output fiber of COM II. Next to the CLS, a QBH (quartz block holder) connector with 3 m long 20/400 μm delivery fiber is spliced. In the configuration setup, PM I and PM II are power-meters for measurement of the backward reflected light and laser output powers receptively. All the components of the system are continuously cooled with a water-cooling system and aluminum heat-sinks.

## Experimental result and discussion

Different pumping schemes have been examined to achieve a 3.02 kW all-fiber oscillator. Here, the effect of the pumping schemes on the laser efficiency and threshold of TMI is discussed in co, hybrid, counter and bidirectional pumping schemes. In the following section, the authors analytically discuss the behavior of TMI in different pumping schemes and attempt to enhance its threshold.

**Figure 2:**
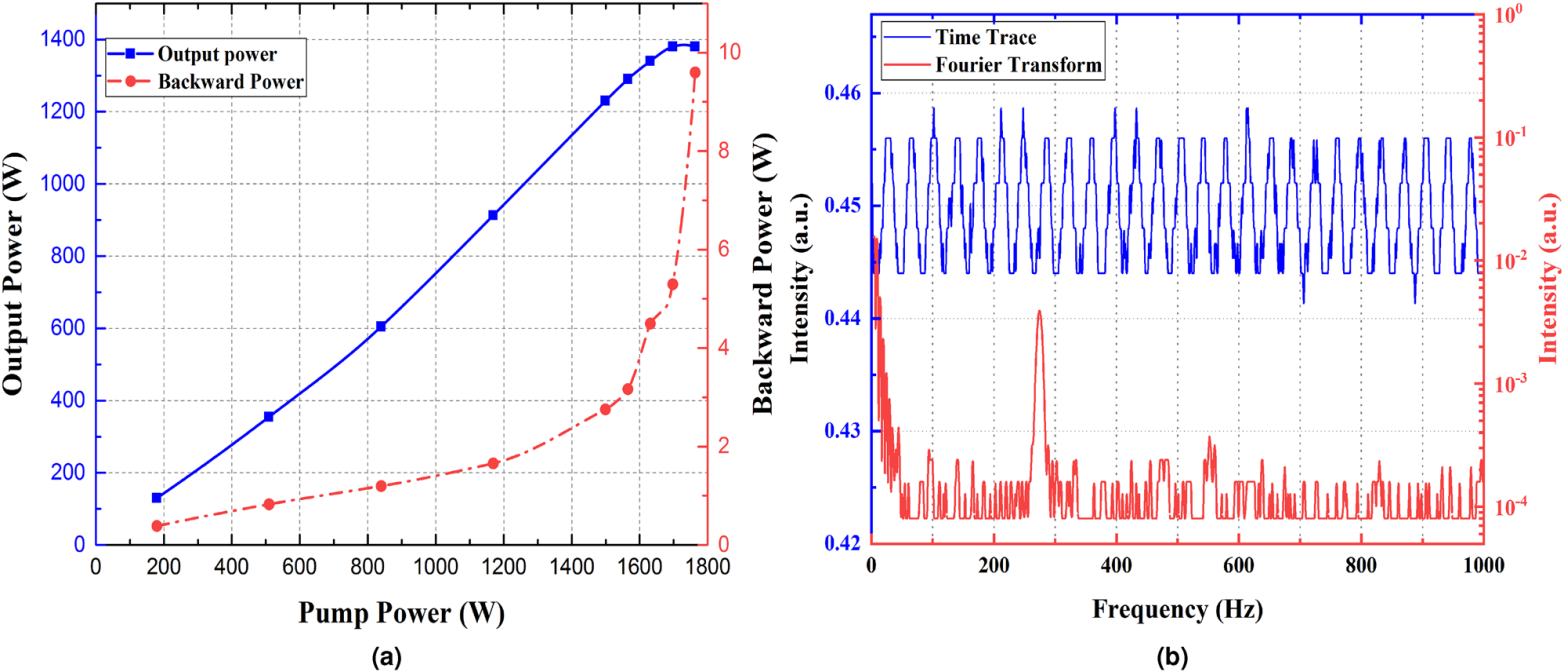
(**a**) The laser output and backward powers versus pumping power in co-pumping configuration. At 1.5 kW pumping power, slope efficiency of the oscillator declines and backward power increases suddenly (**b**) Temporal characteristics of laser output beam at 1.5 kW pumping power

### Co-pumping configuration

In the first experiment, 1800 W pumping power is launched into the laser system in the co direction. The laser output and backward powers versus pumping power are depicted in Fig. 2a. It is observed that at 1500 W pumping power, the laser efficiency declines and backward power starts to increase.

Beyond TMI threshold, energy coupling occurs between the $LP_{01}$ and $LP_{11}$ modes. Then, the portion of $LP_{11}$ mode will increase in modal content in both forward and backward directions. Since the bending loss of $LP_{11}$ mode is much higher than that of $LP_{01}$ mode, the laser output power reduces and optical efficiency declines. The reason for the sharp increase of backward light power beyond TMI threshold backs to the higher transmission of HR FBG for $LP_{11}$ mode^23,24^. Coincidence of these two effects indicates the appearance of TMI^25^. Moreover, M2 factor before and after the TMI threshold is measured. It has been increased from ($M_{x}^{2}=1.3 , M_{y}^{2}=1.2$) to ($M_{x}^{2}=1.7 , M_{y}^{2}=1.9$) when the pumping power exceeds 1500 W which is another evidence of the emergence of TMI in this pumping power. In order to investigate the behavior of TMI in detail, a high-speed photo-detector with 150 MHz bandwidth is employed to monitor the time trace of the laser output beam and calculate its corresponding Fourier spectrum.

Figure 2b shows periodic behavior of time trace in a millisecond timescale above TMI threshold. The first notable peak of fluctuations emerges at 270 Hz, in the Fourier spectrum. Usually, the characteristic frequencies of the time-domain just at the TMI threshold range from 100 Hz to kHz based on the laser parameters and configurations^5,21,26,27^. In amplifiers, most reported frequencies are in the order of kHz and for oscillators, they are usually in the order of hundreds of Hz^28^.

It has been observed that by increasing the pumping power above the TMI threshold featured periodic temporal fluctuation disappeared indicating the appearance of chaotic regime of TMI. For more detailed explanation of the TMI dynamics, the near-field intensity distribution is captured by a high-speed CCD camera with a frame rate of 2000 f/s (see supplementary video 1 which shows the recorded beam profile at the TMI threshold). The media explicitly shows the periodic fluctuations of the fundamental mode intensity on a millisecond timescale. The $LP_{11}$ mode is not clearly seen during the beam fluctuations because of the tight bending of active fiber. The bending diameter of the active fiber is 10–12 cm for which the bending loss of $LP_{11}$ mode is much higher than that of $LP_{01}$. Beyond the threshold of TMI, energy transfer between $LP_{01}$ mode and $LP_{11}$ mode occurs. Due to the higher bending loss of $LP_{11}$ mode, it can slightly be seen in the output beam profile and one can only see the $LP_{01}$ mode intensity fluctuates, while $LP_{11}$ mode is leaked into the cladding and not demonstrated in the media.

### Hybrid pumping configuration

The influence of pump wavelength on TMI threshold has been previously investigated. TMI threshold can be doubled by changing the wavelength of the pump power from 976 to 915 nm^29^. Since the gain fiber required for 915 nm pumping is 3 times longer than that of 976 nm, it leads to the reduction of other non-linear thresholds. Therefore, it is not a good mitigation strategy. Moreover, quantum defect heat load is higher while pumping at 915 nm in respect to 976 nm. It is a better mitigation strategy to use a pumping wavelength that is slightly detuned from 976 nm. For this purpose, to reduce the quantum defect heat load, a 971 nm wavelength stabilized diode laser is employed. At this pumping wavelength, the absorption cross-section is approximately equal to that of 915 nm, but the quantum defect is much lower. Then, it is attempted to investigate the relationship between TMI threshold and gain saturation when pumping by 976 and 971 nm simultaneously. In this regard, one of the 300 W 976 nm LDs in the co-pumping configuration is replaced by a 400 W wavelength stabilized 971 nm LD.

**Figure 3:**
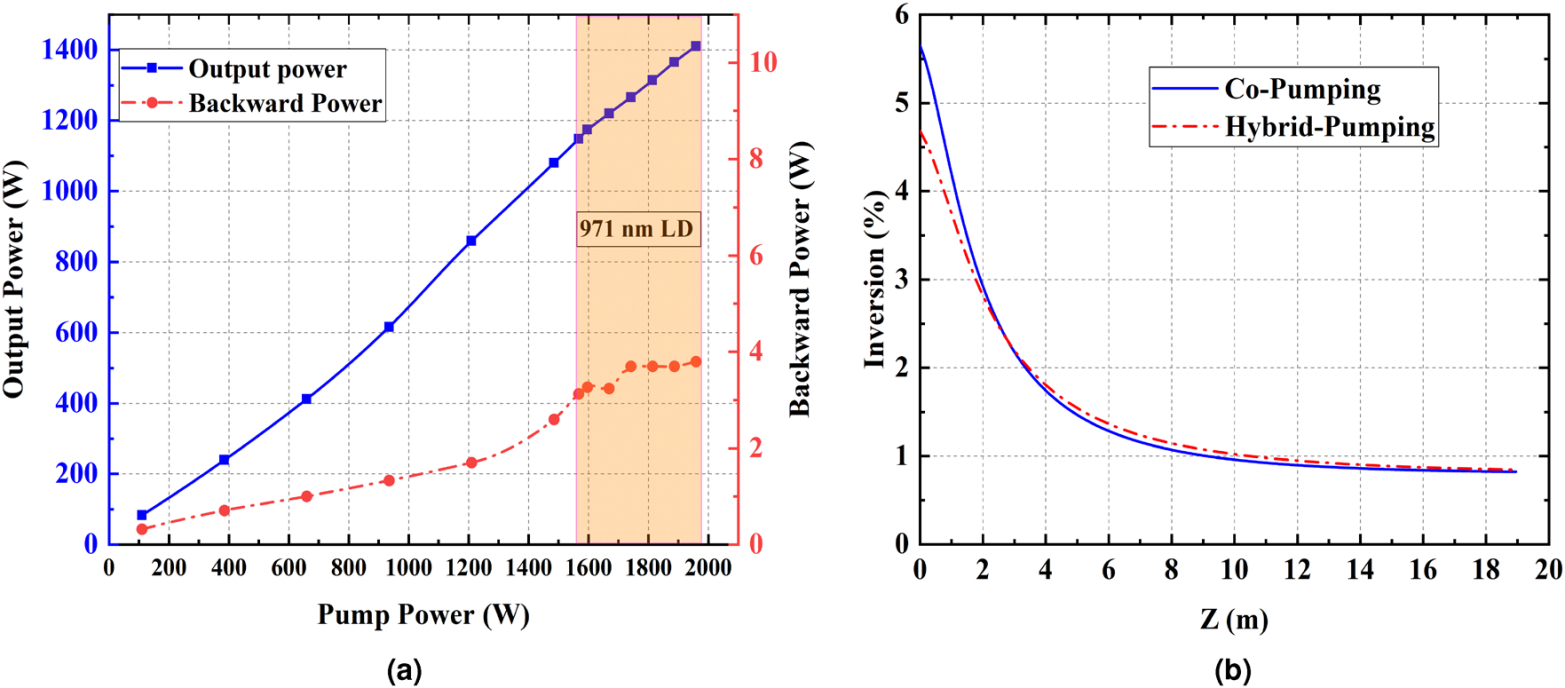
(**a**) Output and backward light powers at different pump powers in the Hybrid pumping configuration. (**b**) Comparison of the co and hybrid pumping inversion level

Figure 3a shows the changes in laser output and backward light powers in terms of pumping power. Interestingly, by increasing the pumping power no sign of dynamic mode coupling in the time trace is observed. Moreover, in comparison of Figs. 3a and 2a, it can be seen that the optical efficiency is increased, and backward light power is decreased in the hybrid pumping configuration. This evidence confirms that TMI is mitigated by hybrid pumping up to 1900 W. In co-pumping, it is observed that TMI occurs at 1500 W pumping power and 1220 W output signal power, but in hybrid pumping, the TMI is not observed up to 1900 W of pumping and 1450 W output powers. Therefore, the TMI threshold has been increased by at least 19% in hybrid pumping. By increasing the total hybrid pumping power higher TMI threshold enhancement is achievable.

It is known that the gain saturation is inversely related to the inversion level. The higher the inversion level, the weaker the gain saturation. The population inversion of co and hybrid pumping schemes were calculated using Liekki application designer V3.3. Figure 3b compares the inversion level as a function of fiber length in co and hybrid pumping schemes. It is shown that the inversion level of hybrid pumping is lower than that of co-pumping. It is worth mention that the quantum defect heat load is higher at the beginning of the active fiber and this region is the most important part for the emergence of TMI. The population inversion at the beginning of the active fiber has decreased by about 21%.

Since the pump absorption coefficient at wavelength of 971 nm and 976 nm is 0.4 and 1.2 dB/m respectively, in hybrid pumping intra-cavity signal propagating in co and counter direction is greater than those of co-pumping configurations. This reduces the inversion level which leads to increase of the gain saturation. Therefore, the threshold power of mode instability can be increased substantially.

### Counter-pumping configuration

In the counter-pumping scheme, 1800 W pump power is injected through COM II (see Fig. 1). The laser output and backward light powers show normal and linear variations as a function of pumping power. In this pumping scheme, the maximum output power of 1450 W and maximum backward light power of 21 W is achieved at 1800 W pumping power while no sign of TMI is appeared in the time trace and its Fourier spectrum.

**Figure 4:**
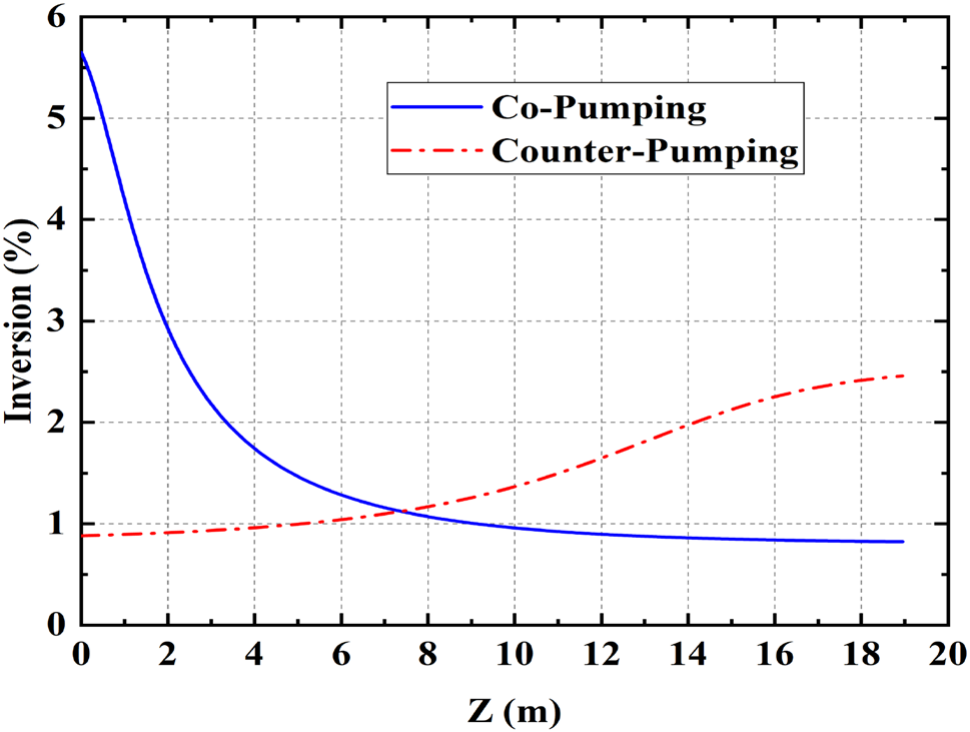
Comparison of co and Counter pumping inversion level

The main difference in the performance of co and counter-pumping schemes roots in different pump and signal distributions along the active fiber. Near the input of the active fiber in co pumping scheme, the pump is strong, and the signal is weak. On the contrary, in counter pumping scheme, signal and pump powers have similar distributions. Based on Ref.^30^, population inversion saturation of these two pumping schemes may play the main role in higher TMI power threshold in counter pumping scheme.

The population inversion of both configurations was calculated using Liekki application designer V3.3. In Fig. 4, the inversion level of co and counter pumping are compared. Obviously, the saturation of the population inversion is much higher in counter-pumping with respect to the co-pumping configuration. The physical cause for the relationship of gain saturation and TMI is that higher gain saturation leads to intenser transversal hole-burning. So the transversal inversion profile looks like a flat-top profile that makes uniform transverse thermal-induced grating. Ultimately as a result of this effect the threshold of TMI enhanced.

Similar to the smith’s theoretical report in fiber amplifiers^30^ our experimental results indicate the substantial role of the gain saturation on enhancement of TMI threshold in fiber oscillators.

**Figure 5:**
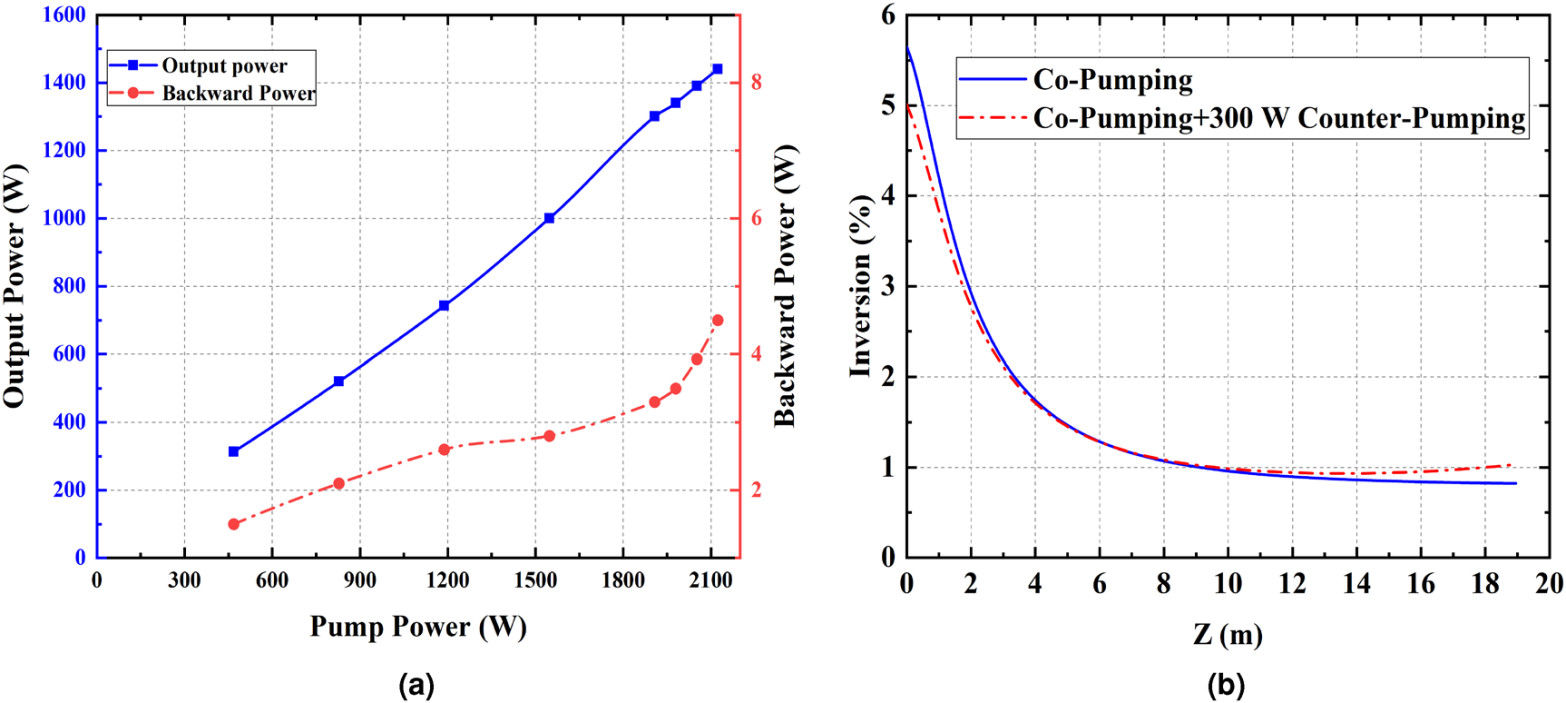
(**a**) Laser output and backward power versus pumping power in bidirectional-pumping setup. Compared with co/counter-pumping setup, The threshold of TMI effect is increased patently (**b**) Comparison of co and bidirectional pumping inversion level

**Figure 6:**
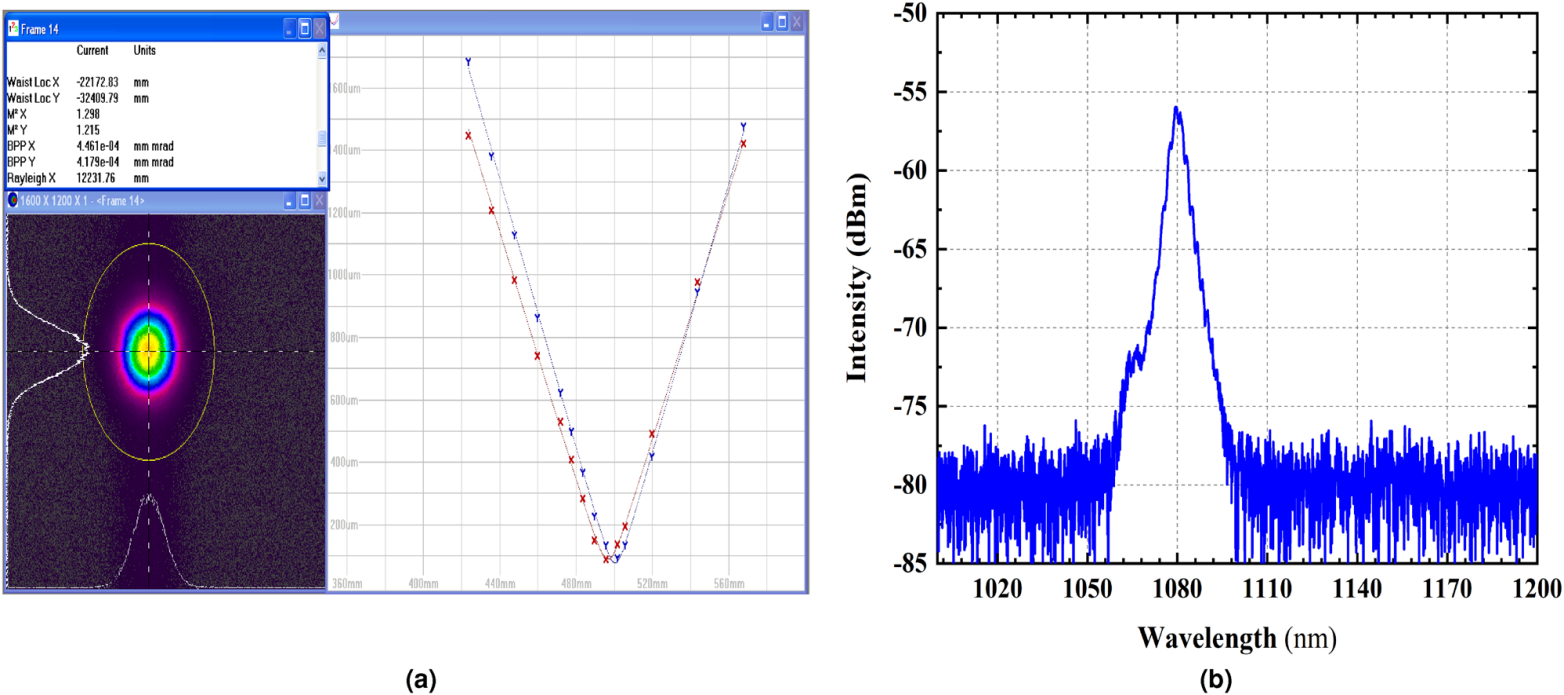
(**a**) The near-diffraction-limited beam quality of the bidirectional-pumping configuration which $M^2 \approx 1.3$ at output power of 3.02 kW indicates the mitigation of TMI. (**b**) spectrum of the fiber laser at 3.02 kW output power

### Bidirectional-pumping configuration

To investigate the effect of population inversion saturation on mitigation of TMI in a bidirectional scheme, the 976 nm co-pumping LDs are turned on along with a counter-pumped LD. In “Co-pumping configuration” section TMI is appears in the co-pumped laser at 1500 W pumping power. Its temporal characteristics are shown in Fig. 2b. It is observed that as soon as a counter-pumping LD turns on, the characteristic frequency in the Fourier spectrum disappears and the backward light power decreases simultaneously. It should be emphasized that this effect has been observed even when the counter-pumping LD is turned on at low power (30 W).

Figure 2a shows the output and backward light powers versus pumping power in the co-pumping scheme. If we compare this figure with Fig. 5a, it is clear that optical efficiency is increased. In this pumping scheme, the pumping power has increased by about 20%, but it is fascinating that the backward power has decreased. Supplementary video 2 shows the recorded screen of the oscilloscope during this experiment which showed that as the counter LD is turned on the periodic shape of the time trace disappeared. Beside the increase of optical efficiency and decrease of backward light power, it is an evidence for mitigation of TMI.

In fact, the interesting result is that in a co-pumped fiber oscillator TMI can be controlled even with very low pumping power from the opposite side. It is observed that the inversion level in this setup is lower than that of the co-pumped scheme. Therefore, injection pump power from the backward direction increases the saturation of population inversion in the bidirectional-pumping configuration (Fig. 5b). Similar to the previous discussions it leads to TMI threshold improvement.

In “Co-pumping configuration” section, it is observed that due to the occurrence of TMI, we were not co-pumped more than 1500 W while using the effect presented here, we were able to pump the highest pump power in this setup (1800 W) after turning on the counter LDs. Ultimately the laser is turned on in maximum available pumping power (3600 W) and 3.02 kW output power is achieved. The time trace and excellent beam quality factor (Fig. 6a), proves that TMI has been controlled in the bidirectional pumping setup. Therefore, TMI is completely mitigated by a bidirectional pumping scheme up to 3.6 kW pumping power and a single-mode beam with M$^{2}$ of $M^2 \approx 1.3$ is achieved. The measured $M^2$-factors and beam profile At the laser operation of 3.02 kW are shown in Fig. 6a.

The optical spectrum of the output laser at 3.02 kW output power is depicted in Fig. 6b. The center wavelength of the output laser operates at 1080 nm and the full width half maximum bandwidth is 5 nm at 3.02 kW. The SRS is not observed at maximum output power.

## Conclusion

TMI behavior in different pumping schemes has been experimentally studied through the fabrication of a 3.02 kW Yb-doped all-fiber oscillator. Comparison of TMI power threshold showed that in hybrid pumping scheme, by choice of 971 nm along with 976 nm pump light, we were able to enhance the co-pumping TMI threshold. By numerical simulation, it is shown that the inversion level of this pumping is reduced. This causes higher gain saturation and enhancement of the TMI threshold. Again, when we inject a few watts of pump power in the backward direction, the backward signal amplifies in the presence of counter pump light. Therefore, this reduces the inversion at the beginning of the active fiber. Our experiments consistently show that population inversion saturation plays an important role in enhancing the TMI threshold in high-power fiber laser systems and are in good agreement with smiths claim in Ref.^30^.

## Supplementary information

Supplementary Video 1.

Supplementary Video 2.
